# Evaluation of Clinical Characteristics and Growth Hormone Response in a Rare Skeletal Dysplasia: Pycnodysostosis

**DOI:** 10.7759/cureus.44823

**Published:** 2023-09-07

**Authors:** Yağmur Ünsal, Suleyman Atar

**Affiliations:** 1 Pediatric Endocrinology, Şanlıurfa Training and Research Hospital, Şanlıurfa, TUR; 2 Pediatric Genetics, Şanlıurfa Training and Research Hospital, Şanlıurfa, TUR

**Keywords:** clinical characteristics, skeletal dysplasia, catch-up growth, recombinant human growth hormone treatment, pycnodysostosis

## Abstract

Introduction

Pycnodysostosis is a rare osteosclerotic skeletal dysplasia; its clinical features include short stature, characteristic facial features, increased bone fragility, and acro-osteolysis of the distal phalanx. Lack of clear guidelines for treatment and follow-up in rare diseases such as pycnodysostosis with growth hormone (GH) deficiency poses a difficulty for the clinician. This study aims to identify clinical, radiological, and endocrine findings of patients with pycnodysostosis focusing on the first year of recombinant human growth hormone (rhGH) treatment response. The eminence of this study is that it presents clinical experience with rhGH, providing an approach for future similar cases.

Methods

Three girls and two boys from three different families diagnosed with pycnodysostosis via clinical, radiological, and genetic evaluation followed up in the pediatric endocrinology clinic between 2022 and 2023 were enrolled in this study. Clinical findings, anthropometric measurements (weight, height, body mass index [BMI]), and laboratory, radiological, and genetic examinations were evaluated retrospectively. Participants were evaluated for GH deficiency using L-DOPA and clonidine tests if growth rate was below -2 standard deviation score (SDS) for gender and age after one-year follow-up.

Results

Complaints on admission were short stature (80%) and recurrent bone fractures (20%). Characteristic facial features and brachydactyly were seen in all the patients. Median height SDS on admission was -3.0 (range: -1.9 to -3.8). Median height SDS on last clinic visit was -3.2 (range: -1.7 to -4.2) at a median age of 8 years (range: 3.5-14 years). BMI was normal in four patients, while one was overweight. Bone mineral densitometry z-score was high, and two patients had bone fractures following minor trauma, while one had recurrent fractures.

Two siblings (first and second cases) and the third case were diagnosed with GH deficiency, and anterior pituitary hormones were normal otherwise. One had partial empty sella in hypophyseal magnetic resonance imaging. rhGH (33 mcg/kg/day, subcutaneously) was started. Growth rate of the first, second, and third cases increased from 3.3, 3.1, 3.9 to 5, 4.3, 7.2 cm/year, respectively. Prior to rhGH, two had adenoid hypertrophy which was stable following rhGH. Growth rate follow-up of the fourth case continues, while the fifth case, the only participant who has reached adult height, has normal height according to age and gender normative.

Conclusion

Although rare, pycnodysostosis should not be overlooked in a patient with characteristic facial features, disproportionate short stature, and recurrent fractures. GH deficiency should be evaluated early if growth rate is declining. rhGH may restore growth rate and the possibility of catch-up in growth in patients with pycnodysostosis and GH deficiency. Hence, after first year of rhGH, growth rate of patients with pycnodysostosis is lower when compared to other etiologies of GH deficiency.

## Introduction

Pycnodysostosis is a rare, autosomal recessive osteosclerotic skeletal dysplasia. First described in 1962, clinical features include short stature (100%), osteosclerosis (100%), recurrent bone fractures (70%), characteristic facial features (midfacial retrusion [frontal bossing, maxillary hypoplasia, small chin, obtuse mandibular angle]), delayed closure of cranial sutures (67%), and acro-osteolysis of the distal phalanx (90%) [1-3]. Located on 1q21, biallelic pathogenic loss of function mutations of cathepsin K gene (*CTSK*) leads to pycnodysostosis [3,4]. Disease prevalence is one to five cases per 1,000,000, where less than 500 individuals with pycnodysostosis have been currently reported [4]. Diagnosis may be made clinically and/or radiologically and confirmed by genetic testing [1,4].

*CTSK* encodes cathepsin K protein, a lysosomal cysteine protease, which is mostly expressed in osteoclasts. Individuals with pycnodysostosis are deficient in cathepsin K. Although the number of osteoclasts is normal, they are functionally insufficient and organic bone matrix proteins secreted during resorption cannot be degraded properly. As a result, demineralized bone tissue is accumulated [5]. Organic bone matrix is not sufficiently degraded, and the old bone material is not resorbed properly, causing the patients to be prone to bone fractures [4]. Phenotypic variability in individuals with pycnodysostosis is known, but a genotype-phenotype correlation in terms of radiological and endocrine features has not been reported [4]. Currently, the treatment for pycnodysostosis is symptomatic with environmental or occupational modifications, orthopedic management for fractures, craniofacial management for cleft palate, maxillary and mandibular hypoplasia, dental anomalies, neurosurgical intervention for craniosynostosis, and pulmonology for obstructive sleep apnea syndrome (OSAS) [5,6].

Although pycnodysostosis is a skeletal dysplasia, and short stature is mainly attributed to skeletal features, growth hormone (GH) deficiency was described in 45% [5]. The pathophysiology of GH deficiency in individuals with pycnodysostosis is yet to be defined, but management with recombinant human growth hormone (rhGH) has been reported. As in other rare diseases, an obstacle for the clinician is that there are no clear guidelines for the optimum timing to investigate for GH deficiency, initiate treatment, and how to follow up. Knowledge on GH deficiency in individuals with pycnodysostosis depends largely on case series, and data on the efficacy of rhGH are scarce. As it is known that growth response after first year of rhGH is the most important predictive factor of overall treatment success [7], it was aimed to explore clinical, radiological, and endocrine features of individuals with pycnodysostosis focusing on rhGH response in the first year. It is expected that a meticulously written approach would offer significant clinical understanding and foundation to upcoming contributions in pycnodysostosis management, as well as contribute to having a better understanding of genotype-phenotype correlation, and underlining radiological and endocrine features.

## Materials and methods

Participants

This study is a retrospective descriptive study. Individuals with pycnodysostosis diagnosed via clinical and radiological evaluation and genetic testing who were followed in the Pediatric Endocrinology Division, Şanlıurfa Training and Research Hospital, were included. Inclusion period was February 28, 2022, to July 30, 2023. Five patients (three girls and boys boys from three different families) with pycnodysostosis were enrolled. Patient records were retrospectively analyzed.

Evaluation on admission and follow-up

The participants were evaluated for medical history, complaints, and clinical findings on admission. Anthropometric measurements (weight, height, body mass index [BMI]) were taken using a Harpenden stadiometer and evaluated using percentile curves standardized for age and gender. Diagnostic laboratory examination as well as radiological evaluation were conducted on admission and were repeated if indicated by disease symptoms and complications. CTSK analysis was conducted following a detailed clinical and dysmorphological examination. Follow-up was conducted every six months for complaints and pycnodysostosis-related complications, as well as anthropometric measurements.

Growth rate of the participants was followed for one year. If growth rate was below -2 standard deviation score (SDS) for gender and age, the participants were evaluated for GH deficiency. Considering the age of our patients, L-DOPA and clonidine tests were used. The tests were conducted according to the standard test protocols [8,9]. Participants who were started with rhGH (33 mcg/kg/day, subcutaneously) were evaluated every three months for growth rate and rhGH dose.

Biochemistry and dual-energy X-ray absorptiometry scan

Routine laboratory examination (complete blood count, biochemistry, blood gas analysis, urine analysis) was conducted every six months following overnight fasting. GH was measured via the radio-immunometric method using Roche Modular E170 Immunology Analyzer (Roche Diagnostics, Turkey, Sanlıurfa), while insulin-like growth factor 1 (IGF-1) was measured using chemiluminescence immunoassay by Siemens IMMULITE 2000 XPi (Siemens Healthcare Diagnostics, Turkey, Ankara). Bone mineral density (BMD) was measured using dual-energy X-ray absorptiometry scan at the lumbar spine (L1-L4). Z-scores were calculated as the difference between the measured BMD and the age- and gender-specific mean value of the population expressed.

Statistical analysis

SPSS Version 28 (IBM Corp., Armonk, NY, USA) was used for statistical analysis. The variables were analyzed for normal distribution using the Shapiro-Wilk test. As the data were not distributed normally, descriptive data were expressed using median (minimum-maximum value), number of patients, percentage, and ratio. Local ethics committee approval as well as written consent from the legal guardians of the patients were obtained.

## Results

Clinical characteristics

All of the patients were of Caucasian descent, with a median age on admission of 3.7 years (range: 2.6-6.2 years) and time from admission to diagnosis of six months (Table 1). Primary complaint was short stature (4/5), while one presented due to recurrent bone fractures.

**Table 1:** Clinical, genetic, and radiological findings of patients with pycnodysostosis. + denotes the presence of feature, - denotes absence of feature F, female; M, male; rhGH, recombinant human growth hormone treatment

Case	1	2	3	4	5
Gender	F	F	M	M	F
Age on admission (years)	3.5	6.2	3.7	2.5	6
Age of diagnosis (years)	4	6.5	4.2	2.5	10
Age at last clinic visit (years)	5.5	8	8	3.5	14
*CTSK* mutation	c.505 G>A p.D169N	c.505 G>A p.D169N	c.3 G>A p.Met1Ile (rs778368118)	c.3 G>A p.Met1Ile (rs778368118)	p.Gly146Arg (c.436G>C)
Consanguinity	+	+	+	+	-
Characteristic facial features	+	+	+	+	+
Frontal bossing	+	+	+	+	+
Obtuse mandibular angle	+	+	+	+	+
Narrow palate	+	+	+	+	+
Delayed closure of the fontanelle	+	+	-	-	+
Delayed teeth eruption	+	+	+	-	-
Dental cavities	-	-	-	-	+
Adenoid hypertrophy	+	-	+	-	-
Brachydactyly	+	+	+	+	+
Dysplastic nails	-	-	-	-	+
Acro-osteolysis of the distal phalanx	+	-	+	+	+
Back pain	-	-	-	-	+
Bone fractures	1	0	0	0	8

**Figure 1:**
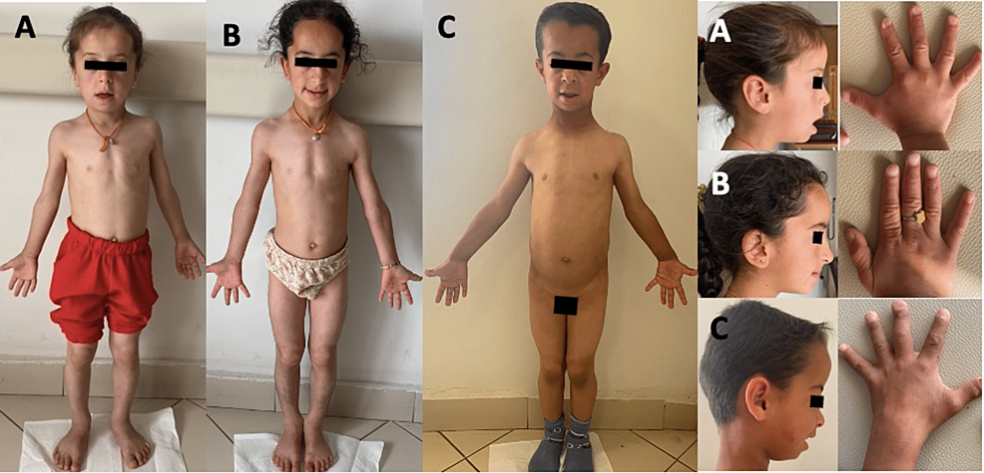
A. Phenotypic features of the first case. Frontal bossing, maxillary hypoplasia, obtuse mandibular angle, disproportionate short stature, and brachydactyly can be noted. B. Phenotypic features of the second case. Notice prominent forehead, obtuse mandibular angle, and disproportionate short stature. C. Phenotypic features of the third case. Frontal bossing, maxillary hypoplasia, obtuse mandibular angle, disproportionate short stature, and brachydactyly are evident.

The siblings (first and second cases) were brought due to short stature. On physical examination, short stature was disproportionate, and characteristic facial features and brachydactyly were evident (Figure 1). While clinical and radiological findings led to the diagnosis, genetic tests confirmed pycnodysostosis. The third case presented due to dysmorphic facial features. On physical examination, he had a disproportionate short stature. After he was diagnosed, at the age of 4.2 years, his brother (fourth case) was examined via family screening. The fifth case was being followed in another clinic for recurrent bone fractures. Osteogenesis imperfecta was suspected; hence, mutation in *COL1A1* and *COL1A2* were not detected. When she was referred to our clinic, and characteristic facial features, brachydactyly, osteosclerosis and acro-osteolysis of the distal phalanx brought up pycnodysostosis, genetic tests confirmed the diagnosis.

All the patients had frontal bossing, obtuse mandibular angle, and narrow palate. Delayed closure of the fontanelles was present in 60%. Brachydactyly was present in all our cases. Dental anomalies were recorded to be 80% (tooth eruption was delayed in 3/5 and cavities was present in 1/5) (Table 1).

Growth and rhGH treatment

Median height SDS on admission was -3.0 (range: -1.9 to -3.8) and median height SDS on the last clinic visit was -3.2 (range: -1.7 to -4.2), with a median age of 8 years (range: 3.5-14 years) (Table 2). Disproportionate short stature was pronounced in 80%. BMI of four cases were normal, while the fifth case was overweight (BMI: 1.7 SDS). Normal pubertal timing was recorded in the fifth case who was Tanner stage 5. Other cases were prepubertal.

**Table 2:** Change in anthropometric measures of five patients with pycnodysostosis during follow-up, rhGH response and IGF-1 measurements in the first, second, and third case, and BMD z-score on admission. rhGH, recombinant human growth hormone treatment; A, on admission; B, before rhGH; SDS, standard deviation score; BMI, body mass index; IGF-1 insulin-like growth factor-1; BMD, bone mineral density + denotes presence of feature, - denotes absence of feature

Case	1			2			3			4		5	
	rhGH			rhGH			rhGH						
Anthropometric measures	A	B	First year	A	B	First year	A	B	First year	A	Last visit	A	Last visit
Height (cm)	88.8	92.1	97.1	98.2	101.3	105.6	88.1	103.0	110.2	80.1	87	126.2	149.2
Height SDS	-2.5	-3.7	-3.2	-3.8	-4.2	-4.2	-2.7	-3.6	-3.3	-3.3	-3.2	-1.9	-1.7
BMI SDS	-0.1	-0.1	-0.1	-0.4	-0.2	-0.2	-1.0	0.2	0.3	0.1	0.2	1.7	1.8
IGF-1 (ng/mL)	48.3		112.1	77.5		157.2	42.2		155.8	38.3		-	
IGF-1 SDS	-3.1		-0.1	-1.1		1.1	-2.1		1.2	-1.7		-	
BMD L1-L4 z-score	2.9			4.5			3.3			-		2.9	
BMD L1-L4 z-score adjusted for height	4.7			6.0			4.8			-		3.9	

All of the patients were evaluated for accompanying etiologies of short stature. History of recurrent infections was absent, and routine biochemical examination as well as inflammatory markers did not reveal any pathological finding. Coeliac disease was ruled out.

**Figure 2:**
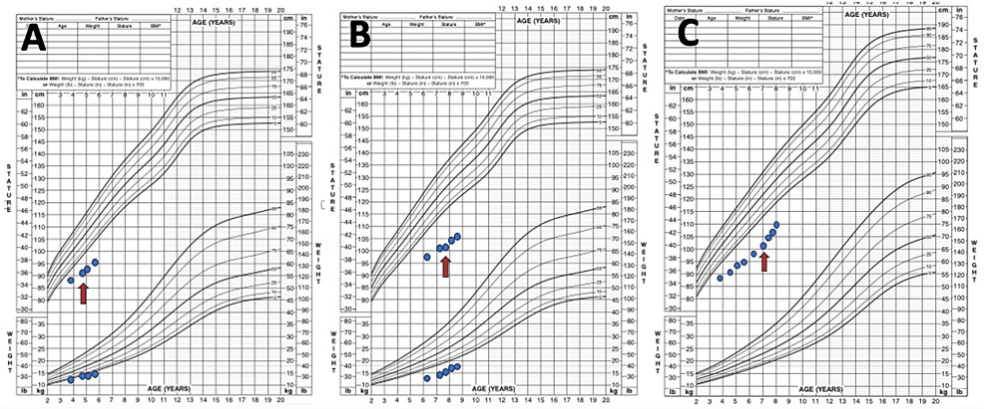
A. Growth chart of the first case who received rhGH. B. Growth chart of the second case who received rhGH. C. Growth chart of the third case who received rhGH. Red arrow represents initiation of rhGH. rhGH, recombinant human growth hormone treatment

One-year follow-up revealed that growth rate of the first, second, and third cases was below -2 SDS for gender and age, and they were evaluated for GH deficiency (Figure 2). GH stimulation tests (GHSTs) of the first case revealed complete GH deficiency (peak GH: 2.8 ng/mL). Hypophyseal magnetic resonance imaging (MRI) was compatible with partial empty sella. Anterior pituitary hormones were normal otherwise. Prior to rhGH, her height was 92.1 cm (-3.7 SDS), and otorhinolaryngology examination revealed mild adenoid hypertrophy. Following a year being compliant to treatment, her height was 97.1 cm (-3.2 SDS), adenoid hypertrophy was stable, and polysomnography was planned (Table 2).

GHSTs of the second case were consistent with partial GH deficiency (peak GH: 7.6 ng/mL). Hypophyseal MRI did not reveal pathological findings, and other anterior pituitary hormone deficiencies were ruled out. Prior to rhGH, her height was 101.3 cm (-4.2 SDS), and adenoid hypertrophy was not detected on otorhinolaryngology examination. Height of the compliant patient was 105.6 cm (- 4.2 SDS) after a year of rhGH (Figure 2, Table 2). Adenoid hypertrophy was absent on repeat examination.

GHSTs of the third case were consistent with partial GH deficiency (peak GH: 8.1 ng/mL). Hypophyseal MRI did not reveal pathological findings, and other anterior pituitary hormone deficiencies were ruled out. His height was 103.0 cm (-3.6 SDS) when he was 7 years old, prior to rhGH. Following a year of rhGH and being compliant with treatment, height was 110.2 cm (-3.3 SDS). Otorhinolaryngology examination before initiating rhGH was consistent with mild adenoid hypertrophy. While findings were stable in last clinic visit, polysomnography was planned. After first year of rhGH treatment, growth rate of the first, second, and third cases increased from 3.3, 3.1, and 3.9 cm/years to 5, 4.3, and 7.2 cm/years, respectively (Figure 2, Table 2). While low prior to treatment, IGF-1 levels were also increased after one year of rhGH treatment (Table 2). Follow-up of his 3.5-year-old brother (fourth case) for growth rate continues.

Radiological findings and bone fractures

**Figure 3:**
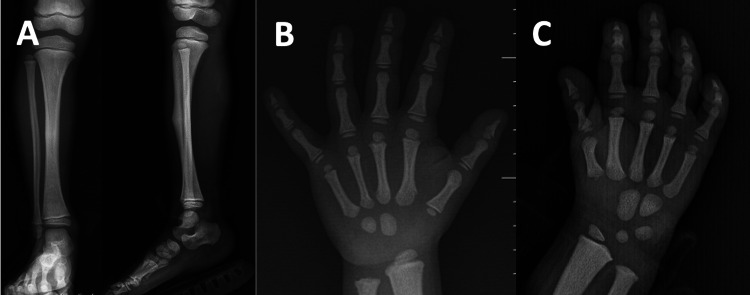
Radiological findings of the first, second, and third cases are being represented. A. Radiological findings of the first case. Osteosclerosis in the tibia and fibula, as well as healing fracture on the tibia can be noted. B. Radiological findings of the second case. Radiograph of the left hand shows osteosclerosis and acro-osteolysis of the distal phalanx. C. Radiological findings of the third case. Radiograph of the left hand shows osteosclerosis and acro-osteolysis of the distal phalanx.

Direct radiography of the left hand revealed acro-osteolysis of the distal phalanx in 80% of the patients. The fifth case had acro-osteolysis of the acromial end of the clavicle. Osteosclerosis was evident in all of the patients (Figure 3). BMD of the lumbar spine (L1-L4) z-score (median: 3.1; range: 2.9-4.5) and height-adjusted BMD of the lumbar spine (L1-L4) z-score (median: 4.8; range: 3.9-6.0) were increased (Table 2). The first case had a fracture in the right tibia after she bumped her leg. First fracture of the fifth case occurred when she was 6 years old following minor trauma. She has had eight fractures in the clavicle, forearm, and tibia, which were due to minor trauma. Her BMD z-score was 2.9. It was observed that bone fractures healed later than normal. Other cases did not have bone fractures.

Comprehensive developmental assessment was performed in the first and fourth cases by a developmental-behavioral pediatrician. Detailed developmental history and Bayley Scales of Infant Development 3rd Edition (BSID-III) of the first and fourth cases were consistent with their peers. [10]. The second, third, and fifth cases were brought to our clinic when they were 6.2, 4.2, and 10 years old, respectively; therefore, BSID-III could not be performed. Detailed developmental history of the second, third, and fifth cases revealed that they had reached developmental milestones within the expected time with school performance consistent with peers.

## Discussion

This study presents clinical, radiological, and genetic findings of five patients with pycnodysostosis from a single center. Evaluation of rhGH response in the first year in three patients was focused on. As acknowledged, lack of clear guidelines for diagnosis, treatment, and follow-up of GH deficiency in rare diseases such as pycnodysostosis poses a difficulty for the clinician. As GH response in the first year is the most important predictive factor of overall treatment success, the study is presumed to provide clinical experience and understanding about rhGH use in patients with pycnodysostosis and would be valuable in providing a step-by-step approach for similar cases to come [7].

Pycnodysostosis is a systemic skeletal disease. Characteristic facial features reflect changes in the cranium (midfacial retrusion [frontal bossing, maxillary hypoplasia, small chin, obtuse mandibular angle]) [5]. All the patients in this study had frontal bossing and obtuse mandibular angle. Delayed closure of the fontanelles was present in 60%. Brachydactyly was present in all our cases, while it reported to be as frequent as 90% in previous studies. Dysplastic clavicles with acro-osteolysis of the acromial end were previously noted in 25% cases [5]. This radiological finding was observed in one (20%) participant. Dental anomalies (delayed eruption of permanent and non-permanent teeth, hypodontia, malocclusion, enamel hypoplasia, predisposition to cavities) were reported to be 30-40% [6,10-12]. In this study, dental anomalies were recorded to be 80%.

Dysplasia of vertebra, especially shortness of the lower extremities in individuals with pycnodysostosis, results in disproportionate short stature. Limbs are often disproportionately shorter than the trunk with rhizo-, meso-, and acromelia [5]. Although intrauterine growth retardation has been reported, short stature commonly develops in early childhood as growth rate declines. Consistent with the literature, 80% of our cases presented because of short stature [13]. Despite disproportionate short stature being mainly attributed to skeletal features, former studies have reported lower circulating IGF-1 levels in pycnodysostosis when compared to other etiologies of short stature. First cases of pycnodysostosis with GH deficiency were reported in 1996; Soliman et al. reported the cases of two children and Darcan et al. reported the case of one child with pycnodysostosis. Following two years of rhGH, growth velocity of these children were improved [14,15]. After that, Rothenbühler et al. reported the cases of three GH-deficient children with pycnodysostosis who also responded well to rhGH [16]. Karamizadeh reported the cases of eight patients with GH deficiency whose growth rate significantly increased after rhGH treatment, with an improvement in height SDS from -4.5 to -3.81 [17]. One of the most comprehensive studies on patients with pycnodysostosis included 27 patients through the French rare diseases Healthcare Network: Bone, cartilage, and calcium diseases. While 45% had GH deficiency and were treated with rhGH, the treatment was efficient in four cases [5]. The pathophysiology of GH deficiency or the results of MRI were not provided in these studies.

The underlying etiology for GH deficiency in patients with pycnodysostosis is yet to be elucidated, but some mechanisms were put forward. Mouse models of pycnodysostosis defined that bone mass and trabecular thickness were increased in sella turcica like other areas of the skeleton, which caused an increase in sellar pressure, resulting in hypophyseal hypoplasia [17,18]. In support of this, clinical studies also reported that individuals with GH deficiency often have pituitary hypoplasia identified on MRI [3,6,14,19]. In cathepsin K deficient state such as pycnodysostosis, the degradation of several matrix-embedded growth factors, including IGF-I, were prevented. IGF-I is released intact with collagen [16]. Although this suggested mechanism speculated that this change in IGF-1 state may alter its local growth-promoting effects, it does not explain GH deficiency encountered in our patients as this explanation displays GH insensitivity.

Low circulating IGF-1 levels and GH deficiency were seen in 60% of our cohort. After a year of growth rate follow-up, GHST of patients with severe short stature was initiated early, as it was proposed that early rhGH treatment restores mid-facial hypoplasia, and decreases mandibular complications as well as OSAS in patients with pycnodysostosis [3,16]. As recommended, cranial MRI was performed in all of the patients; one patient had partial empty sella, a previously non-reported finding. Though this finding may be coincidental as it is frequent in the general population, it may also be an outcome of the previously explained mechanism of increased sellar pressure. As reported in our study, Turan also concluded that deficiency of anterior pituitary hormones other than GH was not seen in individuals with pycnodysostosis and that pubertal maturation was on time [6].

Around 40% of the individuals with pycnodysostosis were reported to have benefited from rhGH [13,16]. The optimum timing for rhGH has not been decided. However, as early rhGH treatment was shown to restore disease features and complications, increasing IGF-1 levels, it is advised to start as soon as the diagnosis is established [3,16]. As being acknowledged, growth response after one year of rhGH treatment is the most important predictive factor of overall success [7]. In this study, following one year of rhGH treatment, growth rate was increased to be near-normal. IGF-1 levels, which are indicative of GH response, were increased, and percentile loss was prevented. It was observed that the increase in growth rate in the first year of rhGH is less pronounced in patients with pycnodysostosis when compared to other etiologies of short stature. Though speculative, this observation may be due to reduced tissue response to rhGH due to skeletal dysplasia. Hence, larger studies with a longer follow-up time are needed, and we intend to collect further data.

The fifth case was the only participant who had reached adult height (149.2 cm). Despite previously reported mean adult height in males (<150 cm; <-2.9 SDS) and females (130-140 cm; <-4.1 SDS), her height was normal according to age and gender normative [13]. Studies concluded that early rhGH enables reaching family’s height potential, near-normalization of adult height, and body proportions, where all the patients were content with treatment [4,12,13,16]. Although obesity is not typical for pycnodysostosis, it was previously described in 26% cases. One of the patients was obese in our study [13].

Severe OSAS requiring noninvasive mechanical ventilation was as frequent as 80% [13,20]. Because of that, as in individuals with Prader-Willi syndrome, it is notified to evaluate individuals for OSAS after diagnosis, prior to rhGH, and during follow-up. As advised, otorhinolaryngology examination was performed prior to rhGH and after the first year. Polysomnography was planned in patients receiving rhGH.

Osteosclerosis, the second most frequent finding, was seen in all of the individuals. It is known that osteosclerosis and impaired microarchitecture of bone result in increased susceptibility to fractures. First fracture is usually seen around 10 years of age [13,21]. The first fracture of the fifth case was following minor trauma when she was six years old. She was admitted due to recurrent bone fractures. This case is important as it underlines that recurrent bone fractures, characteristic facial features, and osteosclerosis should prompt pycnodysostosis while making a differential diagnosis. As observed in our study, healing of fractures may take longer, and remodeling is usually incomplete [22]. A correlation between genotype of BMD and frequency of bone fractures was not observed [4]. Likewise, although the first case had her first bone fracture at 5.5 years, her older sibling (eight years), with the same genotype, have not encountered any fractures.

As encountered in rare diseases such as pycnodysostosis, the number of individuals enrolled in this study was limited, and, thus, a controlled trial and statistical comparison was not possible. Another limitation of the study is its retrospective nature. Some of the important data such as baseline body proportions and their change with rhGH could not be observed, and important developmental assessment on admission could not be performed. However, response to rhGH in three patients with pycnodysostosis was presented comprehensively regarding the rarity of the disease, and that knowledge is based primarily on single case reports and case series. Given the insufficiency of data on the effectiveness of rhGH on individuals with pycnodysostosis for optimal growth and restoration of body proportions, larger studies of international collaborative network with longer follow-up time evaluating adult height are needed. Another future prospect may be determining the criteria for efficiency of rhGH to navigate treatment strategy in individuals with pycnodysostosis.

## Conclusions

Pycnodysostosis, a rare skeletal dysplasia, should be considered if characteristic facial features, disproportionate short stature, and recurrent bone fractures are encountered. Growth rate should be closely monitored in individuals with pycnodysostosis, and GHST should be performed promptly if a decline is detected. rhGH may restore growth rate to be near-normal and prevent percentile loss in individuals with pycnodysostosis and GH deficiency. The increase in growth rate in the first year of rhGH was observed to be less pronounced in individuals with pycnodysostosis than other etiologies of short stature.
